# Smooth muscle α-actin is a direct target of PLZF: effects on the cytoskeleton and on susceptibility to oncogenic transformation

**DOI:** 10.18632/oncotarget.104

**Published:** 2010-05-01

**Authors:** Jin Shi, Minghao Sun, Peter K. Vogt

**Affiliations:** ^1^ Department of Molecular and Experimental Medicine, The Scripps Research Institute, La Jolla, CA 92037

**Keywords:** smooth muscle α-actin, actin cytoskeleton, oncogenic transformation, PLZF, transcriptional repression

## Abstract

Changes in cell morphology and rearrangements of the actin cytoskeleton are common features accompanying cell transformation induced by various oncogenes. In this study, we show that promyelocytic leukemia zinc finger protein (PLZF) binds to the promoter of smooth muscle α-actin, reducing mRNA and protein levels encoded by this gene and resulting in a reorganization of the actin cytoskeleton. In cultures of chicken embryo fibroblasts (CEF), this effect on α-actin expression is correlated with a change in cellular phenotype from spindle shaped to polygonal and flattened. This morphological change is dependent on Ras function. The polygonal, flattened CEF show a high degree of resistance to the transforming activity of several oncoproteins. Our results support the conclusion that reorganization of the actin cytoskeleton plays an important role in tumor suppression by PLZF.

## INTRODUCTION

The cytoskeleton is a filamentous system composed of polymers of actin, tubulin and intermediate filament proteins that provide the framework for nearly all cellular processes. Actin is a core component of the cytoskeleton and plays a vital role in the regulation of cell migration, morphology, tumorigenesis, transport, signaling, cytokinesis, and muscle contraction [1-16]. Actin is the most abundant intracellular protein in the eukaryotic cell and accounts for ~1-5% of the total cell protein in non-muscle cells and ~10% in muscle cells [17]. The vertebrate actin family is comprised of six different isoforms. Four tissue-specific actin isoforms, including skeletal α-actin, cardiac α-actin, smooth muscle α-actin, and smooth muscle γ-actin, are respectively predominant in the adult skeletal and cardiac striated muscles, and in vascular and in enteric smooth muscle [18-20]. Two other actin isoforms are the ubiquitous β- and γ-cytoplasmic actins [20-23]. These actins are highly homologous in animal species from birds to mammals, differing from each other by less than 5% of their amino acid sequence. They are encoded by separate, unlinked genes [21, 22].

The promyelocytic leukemia zinc finger protein (PLZF) is a transcriptional repressor that is characterized by nine C-terminal *Krüppel*-like C2-H2 zinc fingers (ZF) involved in DNA binding and an N-terminal BTB/POZ domain (bric-à-brac, tramtrack, broad complex/poxvirus zinc finger) mediating protein homo- and hetero-dimerization [24]. PLZF is a member of BTB-ZF family of transcription factors. The POZ domain of PLZF recruits several transcriptional corepressors for transcriptional repression [25, 26]. PLZF has multiple functions [27-32], but as a transcriptional repressor, it primarily affects cell proliferation, apoptosis, and differentiation. It regulates cyclin A2 [33, 34], c-Myc [35], c-Kit [36] and other growth-related targets. PLZF is considered to be a tumor suppressor [37-40]. PLZF mRNA expression is a significant predictor of long-term overall survival in malignant melanoma [41].

The cytoskeleton plays a critical role in various cellular processes linked to oncogenic transformation, including proliferation, contact inhibition, anchorage-independent cell growth, and apoptosis [42-46]. Here, we show that overexpression of PLZF in CEF decreases the expression of smooth muscle α-actin and alters cell morphology, inducing resistance to cellular transformation caused by various oncoproteins. The results suggest that the tumor suppressor role of PLZF involves the actin cytoskeleton.

## MATERIALS AND METHODS

### PLZF expression vector

The PLZF expressing vectors, RCAS(B)-Flag-PLZF and pcDNA-Flag-PLZF, have been described [47].

### Viruses expressing oncoproteins

The following retroviruses were used in oncogenic transformation assays: RCAS Myr-P3k (expressing myristylated p110α of chicken phosphoinositide 3-kinase), RCAS Myr-Akt (expressing myristylated chicken Akt-1), RCAS c-Myc (expressing cellular Myc), ASV31 (expressing chicken v-Qin, avian forkhead box G1 protein), AS42 (expressing v-Maf, avian musculoaponeurotic fibrosarcoma oncoprotein), NK24 (expressing v-Fos, Finkel-Biskis-Jinkins murine osteosarcoma viral oncoprotein), ASV1 (expressing v-Crk, chicken tumor 10 oncoprotein), RCAS v-Abl (expressing Abelson murine leukemia viral protein tyrosine kinase), PR-RSV(A) (expressing v-Src, viral Src protein of the Prague strain Rous sarcoma virus), YSV (expressing v-Yes, Yamaguchi sarcoma viral oncoprotein), and ASV17 (expressing v-Jun, the viral Jun oncoprotein) [47]. All viruses expressing oncogenes carried the subgroup A envelope protein, precluding interference with the PLZF-expressing vector which carried the subgroup B envelope protein.

### Culture of CEF for transfection and cellular transformation assays

CEF (chicken embryo fibroblasts) were prepared from White Leghorn embryos obtained from Charles River Breeding Laboratories (Wilmington, MA). CEF were transfected with RCAS(B) vectors expressing Flag-tagged PLZF or vector only by using the Lipofectamine reagent (Invitrogen, Carlsbad, CA). These cultures were tested for their susceptibility to transformation by superinfection with avian retroviruses expressing specific oncogenes. Quantitative determination of oncogenic transformation was performed by enumerating foci of transformed cells (microtumors) caused by the superinfecting virus on the CEF monolayer. For this purpose, cells were stained with 2% crystal violet in 20% methanol.

### Western Blotting

Western blotting and Northern blotting were done as described [47]. Lysates containing 20 μg of protein were separated by SDS-PAGE and transferred to Immobilon-P membranes (Millipore, Billerica, MA). The membranes were then probed with the following primary antibodies: anti-α-actin (Sigma, St. Louis, MO), anti-β-actin (Cell Signaling Technology, Beverly, MA), anti-Flag M2 (Sigma, St. Louis, MO), and Anti-α-tubulin (MP Biomedicals Inc., Solon, OH). After incubation with secondary HRP (horseradish peroxidase) conjugated antibodies (Pierce, Rockford, IL), the reactive bands were visualized by chemiluminescence using the SuperSignal West Pico Chemiluminescent Substrate Kit (Pierce, Rockford, IL), according to the manufacturer's protocol.

### Reverse transcription-PCR and Northern analysis

Total RNA was isolated from CEF as described [47]. 5 μg of total RNA and 50 ng random primers were used to synthesize cDNA with SuperScript™ II RNase H Reverse Transcriptase (Invitrogen, Carlsbad, CA). PCR was subsequently performed by using cDNA and the following primers: 5'-GTGTGATGGTTGGTATGGG-3' (forward) and 5'-GTCACGGACAATTTCACG-3'(reverse). The 509-bp PCR fragment of chicken smooth muscle α-actin was cloned into the pGEM-T vector (Promega, Madison, WI), resulting in pGEMT-αActin, and sequenced. Northern analysis was performed using 15 μg of total cellular RNAs as described [48]. The following hybridization probes were used: the *Nco*I/*Nde*I 509-bp fragment α-actin of the pGEMT-αActin and the EcoRI insert 1,213-bp fragment of the quail glyceraldehyde-3-phosphate dehydrogenase (GAPDH) cDNA [49]. The probes were labeled with [α-32P] dCTP by using Random Primed DNA Labeling Kit (Roche, Indianapolis, IN).

### Cloning of chicken smooth muscle α-actin promoter and construction of reporter systems

Chicken genomic DNA was isolated from CEF by using the QIAamp DNA Mini kit (QIAGEN, Valencia, CA). The truncated promoter segments of α-actin were generated by PCR amplification using 100 ng of genomic DNA and the following primers designed with *Kpn*I and *Nhe*I (forward) or *Xho*I (reverse) restriction sites:

5'-GGTACCGGGCTGCTCATGAGACACAG-3' (-910);

5'-GCTAGCTTGCTGCATTTTACAAGTTCTGCAG-3' (-257);

5'-GCTAGCTCGACCCAGATTAGAGG-3' (-151);

5'-GCTAGCGGTCCCTATATGG-3' (-122);

5'-CTCGAGAGCTCTGGGATGGTG-3' (+19).

Underlined letters in these primer sequences indicate *Kpn*I, *Nhe*I, or *Xho*I sites. Numbers in parentheses are nucleotide sequence positions relative to the mRNA cap site (+1). The PCR fragments were subsequently ligated into the pGEM-T vector and sequenced, then excised with *Kpn*I-*Xho*I or *Nhe*I-XhoI and cloned into *Kpn*I-*Xho*I or *Nhe*I-*Xho*I sites of the pGL3-basic vector (Promega, Madison, WI), resulting in pGL910, pGL257, pGL151, and pGL122, respectively. The PLZF expression vector, pcDNA-Flag-PLZF, was generated by inserting the SfiI fragment of pBSFI-Flag-PLZF into pcDNA3.Sfi vector [47, 50]. For Reporter assays, CEF were seeded into MP-24-well plates at 8×10^4^ cells per well. On the next day, the cultures were co-transfected with 100 ng of each reporter plasmid and various amounts of pcDNA3-Flag-PLZF expression vector using PolyFect reagent (QIAGEN, Valencia, CA). The total amount of transfected plasmid was kept constant by addition of empty pcDNA3 plasmid DNA. Transfected cells were harvested at 48 h posttransfection, the cultures were lysed in 120 μl of passive lysis buffer and firefly luciferase activities were measured according to the manufacturer's protocol (Promega, Madison, WI). Firefly luciferase activities were normalized according to protein concentrations and are represented as the mean of three independent experiments.

### ChIP assay

CEF transfected with RCAS retrovirus encoding Flag-tagged PLZF or RCAS vector only were cultured as monolayers. Cells were fixed by direct addition to the culture medium of formaldehyde at a 1% final concentration at 37°C for 10 min. Fixed cells were harvested for immunoprecipitation following instructions for the Chromatin Immunoprecipitation (ChIP) Assay Kit (Upstate Biotechnology Inc., Lake Placid, New York) with minor modifications. Cross-linked chromatin was immunoprecipitated with anti-PLZF monoclonal antibody (CALBIOCHEM, San Diego, CA) and bound to protein A agarose beads. Mouse IgG (Santa Cruz Inc., Santa Cruz, CA) was used for control experiments. The precipitated chromatin DNA was then purified by QIAquick PCR purification kit (QIAGEN, Valencia, CA). A 203-bp product, specific for the region from −159-bp to +43-bp of the chicken α-actin gene, was amplified by PCR with primers (forward: 5'-AGGGCCTGTCGACCCAGATTAGAGG-3', reverse: 5'-TGACAGTGCTTGGCTGGGGA-3').

PCR conditions were as follows: 94°C for 1 min, followed by 40 cycles of 94°C for 30 s, 60°C for 30 s, and 68°C for 20 s, and hold at 4°C. PCR products were run on a 2% agarose gel.

### Preparation of Nuclear Extracts

Nuclear extracts from CEF were prepared as described [51, 52]. The cells from a 10 cm dish were trypsinized and harvested by centrifugation, washed with 1 × PBS, and resuspended in hypotonic buffer (10 mM HEPES, pH 7.9, 1.5 mM MgCl2, 10 mM KCl, 0.2 mM PMSF, 0.5 mM DTT). The cells were allowed to swell on ice for 10 min. The nuclei were centrifuged and resuspended in ice-cold low salt buffer (20 mM HEPES, pH 7.9, 25% glycerol, 1.5 mM MgCl2, 0.02 M KCl, 0.2 mM EDTA, 0.2 mM PMSF, 0.5 mM DTT). Subsequently, an equal volume of ice-cold high salt buffer (20 mM HEPES, pH 7.9, 25% glycerol, 1.5 mM MgCl2, 1.2 M KCl, 0.2 mM EDTA, 0.2 mM PMSF, 0.5 mM DTT, 1x protein inhibitor cocktail) was added dropwise with stirring. The resulting suspension was rocked gently for 30 min to allow extraction of nuclear proteins. The nuclei were centrifuged again for 30 min, and the resulting supernatant was dialyzed for an hour against dialysis buffer (20 mM HEPES, pH 7.9, 20% glycerol, 100 mM KCl, 0.2 mM EDTA, 0.2 mM PMSF, 0.5 mM DTT). All buffers contained a protease inhibitor (1x) (Complete EDTA-free protease inhibitor cocktail tablet, Roche Molecular Biochemicals Indianapolis, IN).

### Electrophoretic mobility shift assay (EMSA)

The following oligomers were used as probes in EMSA:

(1) 5'- GTGGAAGGGACTGAGGGCCTGTCGACCCAGATT-AGAGGTT-3';

(2) 5'ATTAGAGGTTTTTGTAATAAGGTCCCTATATGGTTTTGTT-3';

(3) 5'-TGGTTTTGTTAGAGACTTCGGCTCTGTCTCTCTC-ATCTCT-3';

(4) 5'-TCTCATCTCTGCTCCTTGTTTGGGAGGCTGGTGGGAGGAG-3';

(5) 5'-GTGGGAGGAGAAGAGCTGAAGGGGCTATATAACCCTGGTG-3';

(6) 5'-AACCCTGGTGCTTTTGGATACACAGTGCACCAT-CCCAGAG-3'.

The oligonucleotide probes were obtained from nucleotides −172 to +19 of the chicken smooth muscle α-actin promoter. The probes form a continuous series with a 10-nucleotide overlap between probes. Annealed oligonucleotides were end-labeled using γ32P-ATP and T4 polynucleotide kinase (NEB, Ipswich, MA), and purified by using spin columns. Each binding reaction contained 3 μg nuclear extract protein in 20 μl of binding buffer (20 mM HEPES, pH 7.5, 1 mM MgCl2, 10 μM ZnCl2, 10% glycerol, 1 mM DTT, 100 mg/ml BSA, 0.15 μg/μl dIdC), and was incubated on ice for 30 min. 100 × molar excess of unlabeled oligonucleotide competitors or 1 μg of anti-Flag antibodies or control mouse IgG antibodies were added 30 min before the addition of labeled probes. The DNA-protein complexes were resolved by electrophoresis through 4% 0.5 × Tris-borate-EDTA-nondenaturing polyacrylamide gels and autoradiography.

### Immunofluorescence

Cells grown overnight on glass coverslips were washed with PBS and fixed with 3.7% formaldehyde in PBS for 30 min. After an additional wash with PBS, cells were permeabilized with PBS containing 0.1% Triton X-100 for 30 min. They were washed again with PBS, and the coverslips were then incubated with a 50 μg/ml fluorescent phalloidin conjugate solution in PBS for 40 min at room temperature in a humidified container (Sigma, St. Louis, MO). During the last 5 min, 4,6-diamidino-2-phenylindole (DAPI) was added at the final concentration of 2 ng/μl. The coverslips were again washed three times with PBS and mounted on glass slides using Slowfade mounting medium (Molecular Probes, Eugene, OR)

## RESULTS

### Overexpression of PLZF downregulates mRNA and protein levels of chicken smooth muscle α-actin

**Fig. 1:**
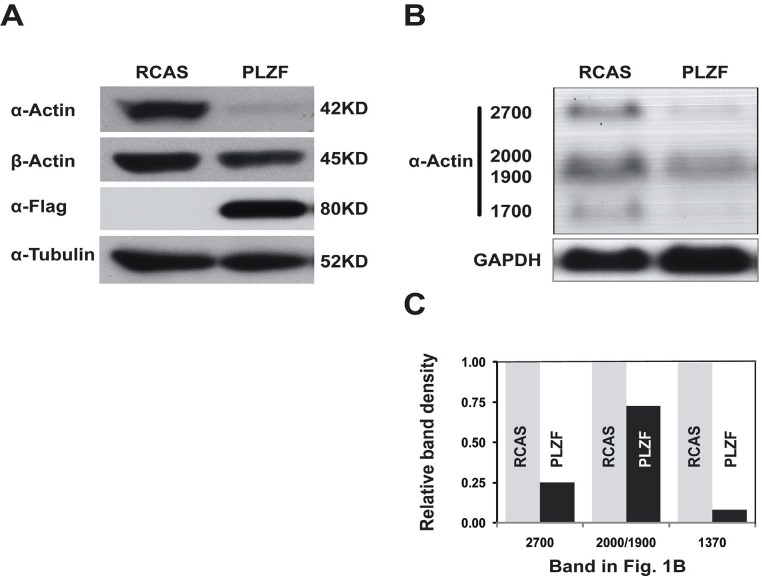
Overexpression of PLZF inhibits the expression of chicken smooth muscle α-actin mRNA and protein (A) Western blot. (B) Northern blot. (C) Quantitative representation of the Northern blot. GAPDH was used as a loading control. CEF were stably transfected with RCAS expressing PLZF proteins or RCAS only.

Overexpression of PLZF from the RCAS retroviral vector in CEF resulted in decreased protein levels of chicken smooth muscle α-actin, but not of β-actin (Fig. 1A). Therefore we examined possible transcriptional repression by PLZF. Total RNA was isolated from CEF expressing PLZF or the RCAS vector only, separated by electrophoresis and examined with the α-actin probe. Northern blots revealed transcriptional repression of chicken smooth muscle α-actin by PLZF (Fig. 1B and 1C). The chicken smooth muscle α-actin gene generates four distinct species of mRNAs with estimated sizes of 1370, 1900, 2000, and 2700 bases. These RNAs differ in the length of their 3' untranslated region, probably as a result of the utilization of alternative polyadenylation signals [53]. PLZF overexpression represses all four mRNAs.

### PLZF downregulates the promoter activity of the chicken smooth muscle α-actin gene

**Fig. 2:**
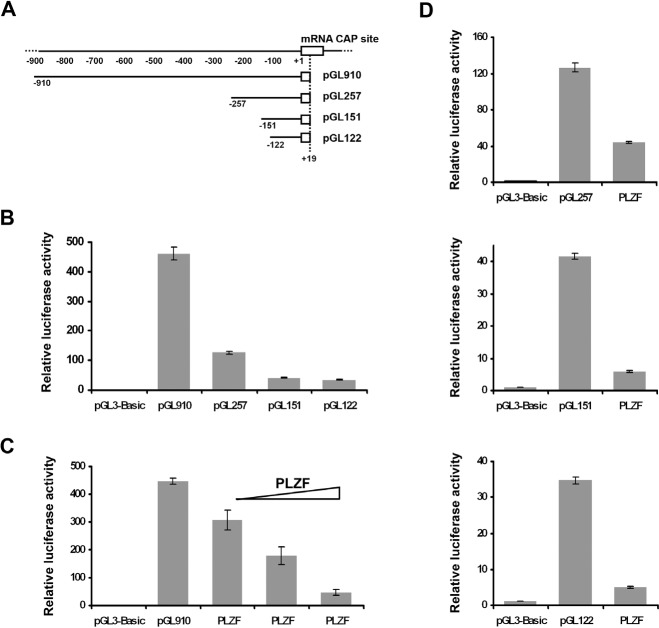
PLZF represses the promoter activity of chicken smooth muscle α-actin (A) Schematic representation of 5' deletion mutations of the chicken smooth muscle α-actin gene. Constructs were prepared using PCR and primers to amplify various portions of the chicken smooth muscle α-actin 5'-flanking region from chicken genomic DNA for subsequent ligation to pGL3-basic vector. The numbers below the diagram of each construct refer to the nucleotide positions of endpoints of deletion mutations, relative to mRNA CAP site at position +1. The names of the corresponding reporter plasmids are shown at the right. (B) Relative transcriptional activities of chicken smooth muscle α-actin promoter deletion mutants. CEF were transfected with equal amount of promoter deletion mutants. (C) PLZF is a transcriptional repressor. CEF were transfected with reporter vector pGL910 along with or without PLZF expression vector pcDNA-Flag-PLZF in the amounts of 3, 30, or 300 ng, respectively. (D) PLZF is able to repress the expression of luciferase activities in various constructs. CEF were transfected with pGL257, pGL151, or pGL122 in the presence or absence of pcDNA-Flag-PLZF. For all assays, cells were harvested 48 hours posttransfection. Relative luciferase activities are normalized by protein concentration and presented as means from triplicate experiments.

Previous studies have delimited the genomic regions that control the transcription of the chicken smooth muscle α-actin gene in CEF and myoblasts [54, 55]. Results of these studies suggest that the first 122 nucleotides 5' of the transcriptional start site function as core promoter and confer high activity to a specific CAT reporter in both fibroblasts and myoblasts. The activity of this core promoter is further regulated by nucleotides −123 to −257 in fibroblasts, with negative regulation mediated by nucleotides −123 to −151. To determine if PLZF is able to bind to the promoter of the chicken smooth muscle α-actin and to function as a transcriptional repressor, we linked the 5' terminus of the promoterless firefly luciferase gene in plasmid pGL3-basic vector to varying lengths of 5'-flanking regions of the chicken smooth muscle α-actin gene, resulting in pGL910, pGL257, pGL151, and pGL122, respectively (Fig. 2A). These promoter deletion mutants were transfected into CEF, and reporter assays were performed to determine whether the 5' flanking sequences of the chicken smooth muscle α-actin were sufficient to direct transcription of the luciferase gene in CEF (Fig. 2B). The reporter plasmid pGL122, containing 122-bp of 5'-flanking sequence, showed the lowest level of transcriptional activity. Addition of 29-bp (nucleotide −123 to −151) in pGL151 slightly increased the transcriptional activity. Addition of another 107-bp (nucleotides −152 to −257) in pGL257 elevated the transcriptional activity further, and pGL910 additionally stimulated the transcriptional activity. Our results show that the activities of promoter deletion mutants were proportional to the length of chicken smooth muscle α-actin 5' flanking region cloned in the reporter constructs (Fig. 2B). All four promoter inserts are able to activate the transcriptional expression of luciferase gene with the longest insert mediating the strongest activation. This result differs from that of previous studies [54], but it is known that the 5'-flanking sequences required for transcriptional expression of the smooth muscle α-actin are highly dependent on cell type [55, 56]. The divergent results could be due to the differences in culture conditions which may favor the prevalence of different cell types [57, 58].

To determine if PLZF is able to act as a transcriptional repressor of α-actin, we overexpressed PLZF in CEF along with the pGL910 which contains the 910-bp α-actin promoter region. In this system, PLZF specifically repressed luciferase activity in a dose-dependent manner (Fig. 2C). It did not repress a β-actin promoter-driven luciferase construct (data not shown). We extended this test to include the pGL257, pGL151, and pGL122 reporters. PLZF downregulated luciferase activity driven by all smooth muscle α-actin promoter constructs (Fig. 2D).

### PLZF binds to the chicken smooth muscle α-actin promoter *in vivo* and *in vitro*

**Fig. 3:**
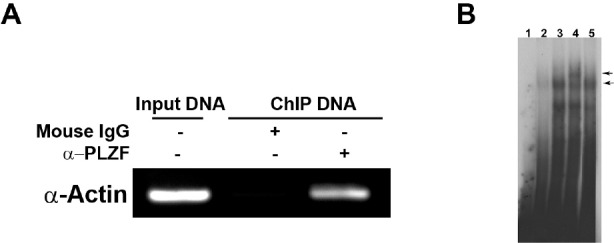
**(A)** PLZF binds to the promoter of chicken smooth muscle α-actin gene in vivo. Chromatin was immunoprecipitated from CEF expressing Flag-tag PLZF with anti-PLZF monoclonal antibody and preimmune mouse immunoglobulin G (IgG). Input is 10% of total sonicated DNA as positive control. Mouse IgG is served as nonspecific control. **(B)** PLZF binds to the promoter of α-actin gene in vitro. Nuclear extracts from CEF expressing Flag-tagged PLZF were used in EMSA. Probe 1 was labeled with 32P-γ-ATP. The protein-DNA complex was revealed in lane 3. Competition experiment was performed using a 100-fold excess of unlabeled probes 1 in lane 2. Supershift experiments were conducted by addition of anti-Flag monoclonal antibody (lane 4) or mouse IgG (lane 5) as a control. Lane 1 represented the free probe. The specific PLZF-DNA and the supershifted complexes are indicated by arrows.

To determine whether PLZF was recruited to the endogenous chicken smooth muscle α-actin promoter *in vivo*, we performed ChIP assays. We immunoprecipitated PLZF from formaldehyde cross-linked chromatin with PLZF antibody. Guided by the results of reporter assays, we designed primers spanning 203-bp from nucleotides −159-bp to +43-bp of the chicken smooth muscle α-actin promoter and analyzed immunoprecipitated chromatin by PCR. The PLZF binding site was amplified in anti-PLZF but not from control mouse IgG chromatin immunoprecipitates (Fig. 3A).

Combining results from reporter and ChIP assays, we subdivided the 5' flanking region from nucleotides −172 to +19 into six oligonucleotides with 10-bp overlaps, designating the oligonucleotides as probe 1 to probe 6 in the direction from −172 to +19, respectively. We then performed EMSA assays with these probes using nuclear extracts prepared from CEF that express Flag-tagged PLZF (Fig. 3B). The EMSA assays identified probe 1 (position −172 to −132) as the only one of the six probes to bind to PLZF and form a protein-DNA complex (Fig. 3B, lane 3; data not shown for probe 2 to 6). The reaction was competed by a 100-fold molar excess of unlabeled probe 1 (Fig. 3B, lane 2). Supershift assays with mouse anti-Flag antibody demonstrated the presence of PLZF protein in the complex (Fig. 3B, lane 4); control mouse IgG did not induce a shift (Fig. 3B, lane 5).

### PLZF induces a reorganization of the cytoskeleton

**Fig. 4:**
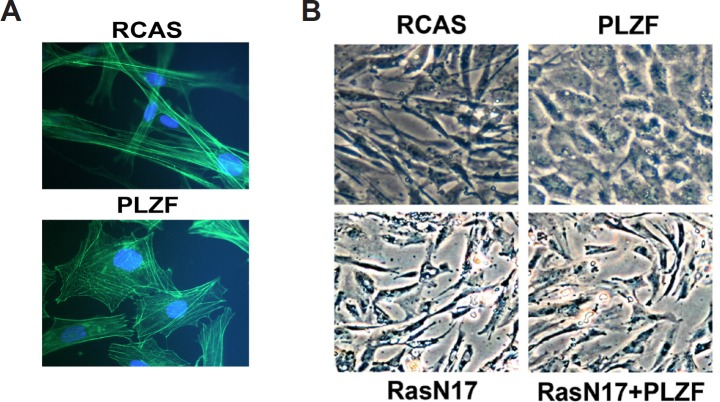
PLZF mediates morphologic change through the Ras pathway **(A)** Morphology and organization of the actin cytoskeleton of CEF stably transfected with RCAS-PLZF or with RCAS empty vector. F-actin is visualized with FITC-conjugated Phalloidin. Nuclei are stained with DAPI. **(B)** Ras activity is required for PLZF-mediated cytoskeleton reorganization. Morphology of CEF transfected with RCAS vector, RCAS(B)-PLZF, RCAS(A)-RasN17, or co-transfected with RCAS(B)-PLZF and RCAS(A)-RasN17 are shown in phase-contrast, demonstrating dominance of the RasN17 phenotype.

Actin exists either as a monomer (G-actin) or as a helically twisted double filament (F-actin) consisting of thousands of actin monomers. F-actin can be found in bundles, called stress fibers, or as fine network underneath the plasma membrane, called microfilaments. In non-muscle cells such as fibroblasts, stress fibers consist of highly organized contractile bundles of actin filaments and bipolar myosin filaments [59-61]. The cellular protein polymer meshwork allows cells to sustain their typical morphology. Actin is a major determinant of cell shape [9, 16, 62, 63]. We investigated the effect of overexpressing PLZF on the morphology of CEF which produce large amounts of smooth muscle α-actin (approximately 10% of the total cell protein) [23]. CEF were stably transfected with RCAS expressing PLZF or with RCAS only, and stress fibers were visualized by labeling with fluorescent phalloidin (Fig. 4A). In the vector-only control, the stress fibers were organized in parallel bundles along the long axis of the cell, resulting in the spindle-shaped morphology characteristic of fibroblasts. In CEF expressing PLZF, stress fibers were shortened and spread in various directions, resulting in a polygonal cell shape. Cells expressing PLZF were also flattened compared to RCAS control cells. These results suggest that PLZF reorganizes the actin cytoskeleton. Whether the downregulation of smooth muscle α-actin by PLZF is the direct cause of this reorganization remains to be investigated.

### The PLZF-induced reorganization of the cytoskeleton and downregulation of α-actin are Ras-dependent

**Fig. 5:**
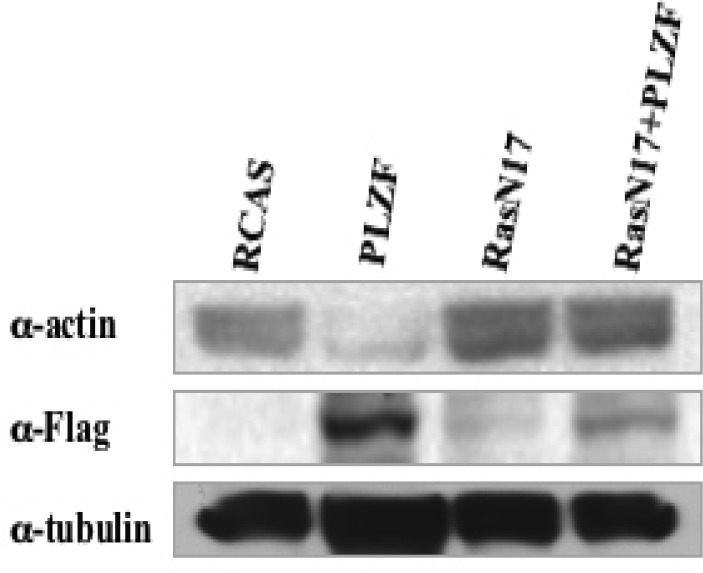
Western blot demonstrating the effect of the RasN17 mutant on the PLZF-mediated reduction in the expression of α-actin. In cells that express RasN17, PLZF fails to downregulate α-actin.

Ras is a GTP-binding molecule that controls several pathways important in cell function. One of these is the Rac/Rho pathway that regulates the actin cytoskeleton [64]. We explored a possible role of Ras in the reorganization of the actin cytoskeleton with the dominant negative Ras mutant Ras S17N (RasN17). This mutant interferes with the function of wild-type, but not oncogenic, Ras [65]. We overexpressed RasN17 in CEF alone or in combination with RCAS or PLZF. CEF expressing RCAS alone showed characteristic fibroblast morphology, and PLZF induced the polygonal, flattened phenotype (Fig. 4B). RasN17-expressing CEF remained fibroblastic but developed numerous vacuoles. In combined expression of PLZF and RasN17, the RasN17 phenotype was dominant. This observation suggests that RasN17 can block the PLZF-mediated rearrangement of the actin cytoskeleton and that Ras activity is required for this rearrangement. RasN17 also abolished the PLZF-mediated downregulation of α-actin expression (Fig. 5), documenting a correlation between the effect of PLZF on cell shape and α-actin expression.

### Suppression of oncogenic transformation by PLZF correlates with the reorganization of the actin cytoskeleton

**Fig. 6:**
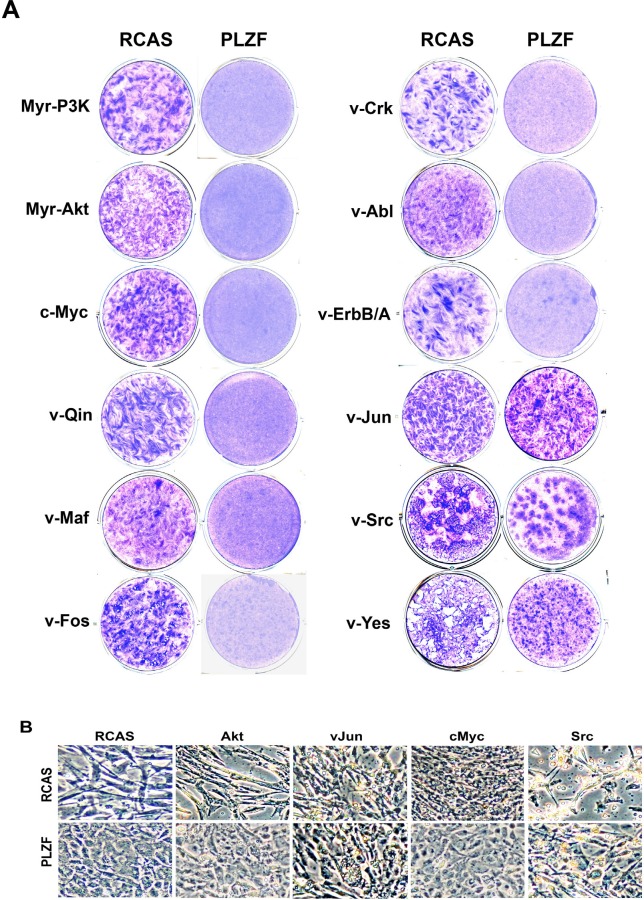
Inhibition of oncogenic transformation by PLZF is correlated with the PLZF-induced change in cell morphology (A) PLZF-mediated interference with oncogenic transformation. CEF stably transfected with RCAS vector expressing PLZF or vector only were superinfected with oncogenic viruses encoding myr-P3k, myr-Akt, c-Myc, v-Fos, v-Qin, v-Maf, v-Crk, v-Abl, v-ErbB, v-Jun, v-Src, and v-Yes. Cells were fed with agar medium and stained after 15 days with crystal violet. (B) The characteristic morphology induced by PLZF is correlated with resistance to oncogenic transformation. CEF stably transfected with PLZF or vector only were superinfected with viruses encoding myr-Akt, v-Jun, c-Myc, and v-Src and cultured in liquid medium. Phase-contrast.

Oncogenic transformation including cell growth, altered cell morphology, anchorage-independent growth, and tumorigenesis is correlated with changes in the cytoskeleton [11, 44, 66-70]. Earlier reports showed that PLZF is growth-suppressive in tumor cell models [24, 33, 71-73]. We had previously found that PLZF interferes with cellular transformation induced by several oncoproteins [47] and have confirmed and extended these data during the present study (Table 1 and Fig. 6A). Our finding that PLZF targets α-actin directly and induces a reorganization of the cytoskeleton suggested a possible mechanism for the interference with oncogenic transformation. We therefore investigated a possible correlation between resistance to transformation and cytoskeletal reorganization. We infected RCAS- or PLZF-expressing cells with viruses encoding various oncoproteins. RCAS-expressing CEF were efficiently transformed by all oncoproteins tested. In contrast, CEF expressing PLZF showed resistance to oncogenic transformation induced by several oncoproteins. Notable exceptions, not affected by PLZF, were v-Src, v-Yes, v-KRas and v-Jun (Table 1 and Fig. 6A).

**Table 1:** Ability of PLZF to interfere with oncogenic transformation

Oncoprotein class	Inhibited by PLZF	Not inhibited by PLZF
Kinases	v-Abl, myr-P3K, myr-Akt, v-Erb-A, v-Erb-B	v-Src, v-Yes
GTPase		v-KRas
Adaptor protein	v-Crk	
Transcription factors	c-Myc, v-Qin, v-Maf, v-Fos	v-Jun

The cellular phenotypes of CEF expressing selected oncoproteins are illustrated in Fig. 6B. Oncoproteins that were inhibited by PLZF did not revert the cytoskeletal reorganization and failed to change the polygonal and flattened morphology of the PLZF-expressing cells. In contrast, the non-inhibited, dominantly acting oncoproteins induced the rounded and swollen cell shape that is characteristically associated with transformation. These data show that the ability of PLZF to act as tumor suppressor is correlated with its cytoskeleton-altering potential and suggest that these cytoskeletal changes are important in blocking oncogenesis.

## DISCUSSION

The observation of downregulated protein expression of smooth muscle α-actin in CEF in the presence of PLZF led us to study how PLZF regulates α-actin. Here we present evidence that PLZF binds to the promoter of smooth muscle α-actin and decreases the expression of mRNA and protein of smooth muscle α-actin. PLZF belongs to the BTB-ZF family of transcription factors that is defined by the presence of an amino-terminal protein-protein interaction domain (BTB-POZ) and carboxy-terminal *Krüppel*-like C2H2 zinc-finger domains. The BTB-ZF family of transcription factors controls a wide variety of biological processes. Two BTB-ZF proteins, scruin and kelch, are involved in regulation of actin cytoskeleton. Scruin is an actin-binding and -bundling protein found in the acrosomal process of *Limulus* sperm [74]. Kelch is a cytoskeletal protein which compacts the actin-based cytoskeleton of the inner rim of the ring canals that are formed during oogenesis in *Drosophila* [75]. The present work shows that smooth muscle α-actin is the transcriptional target of PLZF.

The results on the interaction of PLZF with the α-actin promoter raise two questions. First, probe 1, the α-actin promoter segment that shows PLZF binding in EMSA assays does not contain one of the previously identified binding sequences [24, 34, 72]. However, PLZF is known for the permissiveness of its DNA interactions, it can bind to seemingly unrelated DNA sites [35, 76]. Second, our EMSA assays failed to detect binding of PLZF outside the promoter segment delimited by positions −132 and −172. Yet in reporter assays, PLZF was able to reduce transcription from pGL122 which encompasses only the first 122 positions upstream of the transcription start site. This result suggests that in the cell, PLZF interacts with the α-actin promoter more broadly than can be detected by EMSA assays and that reporter assays are more sensitive for such interactions.

Although smooth muscle α-actin is present predominantly in vascular smooth muscle cells, smooth muscle α-actin is also expressed in CEF [23], which serve here as a model for the changes in cytoskeleton architecture upon PLZF expression. We observed that PLZF-expressing cells undergo significant morphological changes, transiting from a fibroblastic spindle shape to a flat, polygonal shape. Studies of cytoskeletal structures with fluorescence microscopy showed that stress fibers are reorganized from long bundles extending parallel to the long axis of the cell to shorter structures that are aligned in varying directions. The actin cytoskeleton is the main determinant of cell shape. It is in a highly dynamic state with continuous assembly and disassembly of actin filaments, allowing the cell to rapidly change morphology in response to different stimuli [8, 16]. The dynamic properties of F-actin affect many cellular activities, and the basic molecular machinery that mediates actin polymerization, depolymerization and organization into higher-order structures is controlled by more than hundred actin-binding proteins [77, 78]. The Rho family of small GTPases plays a critical role in the regulation of the actin cytoskeleton [79-83]. Rac1 regulates the formation of lamellipodia and membrane ruffles, RhoA induces the formation of stress fibers, and Cdc42 mediates the formation of microspikes and filopodia [84, 85]. Ras functions upstream of the Rac/Rho pathways [64, 86-88]. Here we blocked Ras activity with dominant negative RasN17 and inhibited the PLZF-induced morphologic change in CEF. These results suggest that the PLZF-mediated change in cell morphology is Ras-dependent and that Ras and some of its targets act downstream of PLZF, but more data are needed to confirm this hierarchy.

In a previous work and in the current study, we have determined the sensitivity of PLZF-expressing cells to transformation by various oncoproteins [47]. These oncoproteins include myr-P3K and myr-Akt which are components of phosphoinositide 3-kinase pathway, the transcription factors c-Myc, v-Qin, v-Maf, v-Fos, and v-Jun, the adaptor protein v-Crk, the GTPase v-KRas, and the tyrosine kinases v-Abl, v-ErbB, v-Src and v-Yes. The oncoproteins tested transform cells by distinctly different mechanisms, yet PLZF interferes with representatives of three major functional classes: lipid and protein kinases, the adaptor protein Crk and several transcription factors. This broad spectrum of tumor suppression probably reflects the control of a fundamental cellular function, and the modulation of the actin cytoskeleton by PLZF is candidate for such a function. When PLZF is effective in blocking oncogenic transformation, the cells remain polygonal and flat. When the tumor suppression is overcome, the flat and polygonal phenotype is also changed.

There is no obvious common denominator of the oncoproteins that can overcome PLZF-mediated tumor suppression. Oncogenic transformation induced by Src family kinases and by Ras causes dramatic changes in the actin cytoskeleton and cell shape not seen to that extend with other oncoproteins [89-91]. The resistance of v-Jun to PLZF-mediated tumor suppression is puzzling, especially in view of the fact that PLZF effectively suppresses transformation by v-Fos. Jun-transformed cells have a characteristic, needle-like morphology indicating a significant intervention in the organization of the cytoskeleton. Although Jun is traditionally paired with Fos to form an AP-1 transcription factor complex, there are several other Jun dimerization partners that play a role in oncogenic transformation. These Jun dimerization partners other than Fos have distinct effects on cell growth. Thus, Jun-Fra2 dimers induce anchorage-independence, Jun-ATF2 dimers mediate growth factor-independence [92]. The spectrum of oncogenic AP-1 dimers that mediate Fos-induced oncogenic transformation is distinct from that of Jun and could therefore be more susceptible to the tumor suppressive effects of PLZF.

Like all transcriptional regulators, PLZF has numerous functions and targets which lead to diverse phenotypic changes in different cell types [27-34, 36-40]. The tumor suppressor activity of PLZF probably reflects a complex sub-set of these interactions that cooperatively affect the cellular phenotype. It is unlikely that any single molecular target of PLZF will explain the entire spectrum of antioncogenic cellular changes. In a recent publication, we have analyzed an effect of PLZF on Myc [47]. We confirmed that wild-type PLZF represses Myc transcription [35], although it did not significantly affect Myc protein levels. However, PLZF reduced the phosphorylation of Myc at T58 and S62, presumably resulting in lowered transcriptional activity of Myc. The PLZF-induced posttranslational changes in Myc were correlated with the inhibition of the relevant Myc kinases. PLZF caused an Akt-mediated downregulation of GSK3β and reduced the activity of MEK1/2. These effects on Myc are probably part of the tumor suppressor activity of PLZF, because the expression of PLZF mutants that do not attenuate Myc leads to oncogenic transformation. The current study suggests that targeting α-actin represents another, different tumor suppressive activity of PLZF. There is a correlation between downregulation of α-actin, reorganization of the cytoskeleton and resistance to the action of certain oncoproteins. The downregulation of α-actin by PLZF directly affects a part of cellular organization that plays a key role in oncogenic transformation and appears to be one of the multipronged actions by which PLZF is tumor suppressive.

## Abbreviations used

CEF: chicken embryo fibroblasts
RCAS: replication competent avian leukosis virus with a splice acceptor
BTB/POZ: bric-à-brac, tramtrack, broad complex/poxvirus zinc finger
GAPDH: glyceraldehyde-3-phosphate dehydrogenase
c-Myc: cellular homolog of the avian myelocytomatosis MC29 viral oncoprotein
Myr-Akt: myristylated Akt-1, cellular homolog of the Akt8 murine lymphoma viral oncoprotein
Myr-P3k: myristylated p110α of phosphoinositide 3-kinase
v-Abl: Abelson murine leukemia viral oncoprotein
v-Crk: chicken tumor 10 viral oncoprotein
v-Fos: Finkel-Biskis-Jinkins murine osteosarcoma viral oncoprotein
v-Jun: avian sarcoma virus 17 oncoprotein
v-Maf: avian musculoaponeurotic fibrosarcoma viral oncoprotein
v-Src: viral Src protein of the Prague strain Rous sarcoma virus
v-Qin: avian viral forkhead box G1 protein
v-Yes: Yamaguchi sarcoma viral oncoprotein Fra, Fos-related antigen
ATF: activating transcription factor
